# Psychometric evaluation and Rasch analyses of the German Overall Anxiety Severity and Impairment Scale (OASIS-D)

**DOI:** 10.1038/s41598-023-33355-0

**Published:** 2023-04-26

**Authors:** Thomas S. Hiller, Sabine Hoffmann, Tobias Teismann, Karoline Lukaschek, Jochen Gensichen

**Affiliations:** 1 Institute of General Practice and Family Medicine, Jena University Hospital, Bachstr. 18, 07743 Jena, Germany; 2 Institute for Medical Information Processing, Biometry and Epidemiology, Ludwig-Maximilians-University, Marchioninistr. 15, 81377 Munich, Germany; 3 Mental Health Research and Treatment Center, Ruhr-University Bochum, Massenbergstraße 9-13, 44787 Bochum, Germany; 4 Institute of General Practice and Family Medicine, University Hospital of LMU Munich, Nußbaumstr. 5, 80336 Munich, Germany

**Keywords:** Anxiety, Psychology, Health care

## Abstract

The Overall Anxiety Severity and Impairment Scale (OASIS) is a 5-item self-report measure that captures symptoms of anxiety and associated functional impairments. This study evaluates a German version (OASIS-D) that was administered to a convenience sample of 1398 primary care patients of whom 419 were diagnosed with panic disorder with/without agoraphobia. Psychometric properties were analyzed using classical test theory as well as probabilistic test theory. Factor analyses suggested a unitary (latent) factor structure. The internal consistency was good to excellent. Convergent as well as discriminant validity with other self-report measures was found. A sum score (range 0–20) of ≥ 8 emerged as optimal cut-score for screening purposes. A difference score of ≥ 5 was indicative of reliable individual change. A Rasch analysis of local item independence suggested response dependency between the first two items. Rasch analyses of measurement invariance detected noninvariant subgroups associated with age and gender. Analyses of validity and optimal cut-off score were solely based on self-report measures, which may have introduced method effects. In sum, the findings support the transcultural validity of the OASIS and indicate its applicability to naturalistic primary care settings. Caution is warranted when using the scale to compare groups that differ in age or gender.

## Introduction

With more than 300 million people being affected worldwide, anxiety disorders are the most common psychiatric diagnoses and a leading cause of disability burden^1,2^. These disorders are associated with severe reductions in quality of life and considerable health-economic costs^3–5^. Effective treatment options are available and should be applied once the psychopathology has been detected^6^. At the same time, only a minority of affected individuals receive adequate treatments^7^.

It is important to identify anxiety disorders in primary care where their prevalence is higher than in the general population^8,9^. Many affected individuals present in primary care settings first and tend to have themselves frequently re-examined in internal or emergency wards^10,11^. However, an under-recognition of anxiety disorders in primary care has often been reported^12–14^.

According to current clinical guidelines, the recognition, diagnosis, and initial (behavioral or pharmacological) treatment for patients with anxiety disorders should be carried out in primary care^15,16^. Treatment progress (including the severity of anxiety symptoms) should be monitored by primary care physicians, and patients should be referred to mental health specialists if the interventions offered in primary care prove to be not successful^15,16^. However, there is a shortage of mental health specialists in many (especially rural) areas^17,18^, resulting, for example, in an average waiting time for psychotherapeutic outpatient treatment in Germany of about 19 weeks^19^. Against this background, it seems of utmost importance to further improve the quality of diagnosis and treatment of mental disorders in primary care.

Various self-report instruments for the assessment of anxiety are available^20^. Most of them are relatively lengthy, disorder-specific, or do not capture functional impairments according to which the clinical significance of symptoms should be evaluated. These measures may be of limited utility for busy primary care settings where time-constraints are of concern^21,22^. Measurement tools suitable for primary care should be brief, easy to administer, reliable, valid, and applicable to all anxiety disorders.

The Overall Anxiety Severity and Impairment Scale (OASIS) has been developed in the United States as a self-report measure that (1) assesses the severity of anxiety symptoms as well as associated functional impairments; (2) can be applied to any anxiety disorder, multiple anxiety disorders, or subthreshold anxiety problems; and (3) is brief enough to be effectively used in busy clinical settings as well as epidemiological research^23^. Several studies have evaluated the original English version of the OASIS using samples of college students^23,24^, primary care patients^25^, patients with post-traumatic stress disorder^26^, and psychiatric outpatients^27,28^. Recently, it has been adapted to yield a caregiver-report of youth anxiety and interference^29^. The OASIS has been translated and validated into Japanese^30^, Dutch^31^, Spanish^32^, Persian^33^, and Czech^34,35^.

Previous research has focused on the scale’s (latent) factor structure, reliability, convergent and discriminant validity, cut-scores, measurement invariance, and sensitivity to change. Exploratory and confirmatory factor analyses support a unidimensional factor structure. However, in all but one study^33^ an acceptable model fit could only be reached by allowing the error terms of the first two items to be freely estimated (i.e., a single-factor-model with correlated residuals of items 1 and 2)^24,25,28,30,32,34–36^. In two studies, the best model fit was obtained by additionally allowing a correlation between the residuals of items 1 and 3^27,30^. These findings call into question the unidimensionality of the scale and thus, whether it is statistically valid to sum up the response items to a single test score (which is the usual scoring algorithm).

Throughout the studies, the OASIS showed good to excellent internal consistency, with Cronbach’s alpha ranging from 0.80 to 0.96^23–25,27,30–36^. Studies examining the convergent validity of the OASIS have found moderate to large correlations with other self-report measures of anxiety using clinical^25–28,30–32,36^ or non-clinical samples^23,24,30,33–35^. This evidence for construct validity has been extended by demonstrating the measure’s sensitivity to change after anxiety-specific treatments^26,28,32,36^.

To explore the diagnostic abilities of the OASIS, some studies have derived clinical cut-scores from calculations of receiver operating characteristics (ROC). Most often, sum scores ranging between 7 to 9 were judged optimal for discriminating individuals with anxiety disorders from those without^24,25,28,30,32^. However, other cut-scores ranging from 5 to 15 have also been found^26,35^.

Few studies have investigated the OASIS for measurement invariance, a statistical property that refers to the assumption that identical item responses convey identical information, regardless of who is providing the response. This assumption is implicitly made whenever test results between different groups of respondents are compared^37^. Using confirmatory factor analyses (CFAs), three studies consistently found measurement invariance of the OASIS with respect to gender^28,34,35^. To our knowledge, no study has yet analyzed the OASIS for measurement invariance or local item independence within the framework of probabilistic test theory.

This study presents a German version of the Overall Anxiety Severity and Impairment Scale (OASIS-D) that was administered to a large convenience sample of primary care patients. We evaluated the OASIS-D using statistical analyses based on classical test theory as well as Rasch analyses based on probabilistic test theory. We expected to find comparable results to previous investigations of the OASIS regarding the (latent) factor structure, internal consistency, convergent/discriminant validity, and clinical cut-scores. By means of Rasch analyses, we aimed to explore the scale’s dimensionality, local item independence, and measurement invariance in greater depth.

## Methods

The research reported here was conducted within the scope of an interventional trial^38,39^. Ethics approval was obtained from the ethics committee of the Friedrich-Schiller-University at the Medical Faculty (Jena, Germany; approval no. 3484-06/12). All study procedures were performed in accordance with the ethical standards of Good Clinical Practice^40^ and with the Declaration of Helsinki and its later amendments^41^. Participation in the research was voluntary and subject to informed consent; necessary information was provided to the participants.

### German translation of the OASIS

We performed a translation of the original brief OASIS^24^ following recommendations for the cross-cultural adaptation of self-report measures^42,43^. The original version was translated by two independent German native speakers and back translated by two independent English native speakers. A pilot testing of the pre-final version showed that several patients deemed item 2 (intensity of anxiety) not appropriate when having answered ‘Never’ to item 1 (frequency of anxiety) and had thus been tempted to skip item 2 which would have resulted in missing data points. This led us to a final modification of response options for item 2: Response item ‘None’ was modified to ‘None/No anxiety’ (German: ‘Gar nicht/Keine Angst’). Using a sample of 434 primary care patients, a preliminary psychometric evaluation of the final version was performed^44^.

### Participants and procedure

Seventy-three primary care practices were trained in diagnostics and treatment of anxiety disorders as part of an interventional trial^39^. All practices were in private ownership and participated in standard medical care. Most were single-handed (62%) and located in rural areas (66%).

For recruitment purposes, the primary care practices administered a screening questionnaire to a convenience sample of their patients (henceforth, the total sample). This questionnaire comprised the OASIS-D as well as the panic disorder module of the Patient Health Questionnaire^45^ and asked participants to indicate their age and gender. The total sample consisted of 1398 primary care patients with a mean age of 49.51 years (SD 16.58; range 16–89 years) of whom 914 (65.4%) were female.

Patients showing an OASIS-D sum score of above seven and two or more positive answers to the PHQ panic module were offered a diagnostic interview with the primary care physician who thereby verified the diagnosis of panic disorder with or without agoraphobia (PDA) using validated ICD-10 checklists. To limit the burden on study participants, the presence of other mental disorders was not assessed. Patients from the PDA-subsample (n = 419) were asked to provide more sociodemographic and clinical data using self-report questionnaires (Table 1).

**Table 1:** Sociodemographic and clinical characteristics of patients with panic disorder.

Age in years, mean (SD)	46.16 (14.42)
Female sex, n (%)	311 (74.2)
Years of education, median (IQR)	10 (9, 12)
Work status, n (%)	
Self-/Employed	262 (63.4)
Unoccupied	61 (14.7)
Retired	82 (19.8)
Comorbid agoraphobia, n (%)	315 (75.2)
Comorbid depressive disorders, n (%)	155 (37.0)
Number of panic attacks during past week, n (%)	
0–1	221 (53.3)
2–3	120 (29.0)
4 or more	73 (17.6)
Severity of panic attacks during past week, n (%)	
None or mild	180 (44.0)
Moderate	133 (32.5)
Severe or extremely severe	96 (23.4)
PHQ panic module, median no. of ‚yes ‘-answers (IQR)	4 (3, 4)
OASIS-D, mean (SD)	12.51 (2.78)
OASIS-D retest, mean (SD)	10.61 (3.64)
BAI, mean (SD)	28.21 (12.36)
MI, mean (SD)	2.27 (0.82)
ACQ, mean (SD)	2.07 (0.68)
ASI-3, mean (SD)	36.58 (15.13)
PHQ-9, mean (SD)	11.19 (5.72)
EQ-5D health status, mean (SD)	57.54 (19.68)
PACIC, mean (SD)	6.17 (2.60)
PAM, mean (SD)	39.46 (5.98)

*ACQ* Anxiety Cognitions Questionnaire, *ASI-3* Anxiety Sensitivity Index-3, *BAI* Beck Anxiety Inventory, *EQ-5D* EuroQoL questionnaire (visual analogue scale), *IQR* interquartile range, *MI* Mobility Inventory (subscale ‘alone’), *OASIS-D* Overall Anxiety Severity and Impairment Scale (German version), *PACIC* Patient Assessment of Chronic Illness Care, *PAM* Patient Activation Measure, *PHQ* Patient Health Questionnaire, *PHQ-9* Patient Health Questionnaire (depression module).

### Instruments

#### International diagnostic checklists for ICD-10 and DSM-IV (IDCL)

The IDCL^46,47^ are a set of semi-structured instruments designed for clinician assessment of mental disorders. The primary care physicians used the checklists for panic disorder and agoraphobia during diagnostic interviews with individual patients to determine the presence of these disorders according to ICD-10 criteria^48^.

#### Overall Anxiety Severity and Impairment Scale (OASIS)

The OASIS^24^ assesses the severity of anxiety and associated functional impairments with five items that are rated on five-point Likert-type scales. For the period of the past week, respondents indicate the frequency (item 1) and intensity/severity (item 2) of anxiety or fear, the frequency of behavioral avoidance (item 3), and the extent of anxiety-related interferences in occupational/domestic (item 4) and social (item 5) areas of life. Sum scores range from 0 to 20, with higher values indicating more anxiety and impairments. The German version (OASIS-D) is provided in Supplementary Appendix 1.

#### Patient Health Questionnaire (PHQ)

The PHQ^45,49^ consists of several modules to detect mental disorders according to DSM-IV^50^. The panic disorder module comprises four items capturing diagnostic criteria of panic disorder. Respondents indicate whether each of these criteria did or did not apply to them (‘yes’/’no’) during the past four weeks. The depression module (PHQ-9) consists of nine items to capture symptoms of depressive disorders that are rated on four-point Likert-type scales. Sum scores range from 0 to 27, with higher values indicating a greater severity of depression symptoms. We used Spitzer et al.’s coding algorithm to determine the presence of comorbid depressive disorders^45^.

#### Beck Anxiety Inventory (BAI)

The BAI^51,52^ is a generic measure of anxiety severity. Respondents rate how severely they had been affected by 21 typical symptoms during the past week using four-point Likert-type scales. Sum scores range from 0 to 63, with higher values indicating a greater severity of anxiety symptoms.

#### Anxiety Cognitions Questionnaire (ACQ)

The ACQ^53,54^ is a 14-item measure that captures thoughts about feared bodily sensations and loss of control that typically occur in individuals with agoraphobia. Respondents rate the frequency of these thoughts on five-point Likert-type scales. Mean scores range from 1 to 5, with higher values indicating a higher frequency of anxiety-related cognitions.

#### Mobility Inventory (MI), subscale ‘alone’

The MI^54,55^ is a 27-item measure that captures situations that individuals with agoraphobia typically fear. With the subscale ‘alone’, respondents rate on five-point Likert-type scales how often they avoid each of these situations when being unaccompanied. Mean scores range from 1 to 5, with higher values indicating a higher frequency of agoraphobic avoidance behaviors.

#### Anxiety Sensitivity Index-3 (ASI-3)

The ASI-3^56,57^ is an 18-item measure that assesses the extent of physical, cognitive, and social concerns due to fear of arousal-related sensations (i.e., anxiety sensitivity). Respondents rate how strongly they agree with such concerns using five-point Likert-type scales. Sum scores range from 0 to 72, with higher values indicating greater anxiety sensitivity.

#### EuroQoL Questionnaire (EQ-5D)

The EQ-5D^58,59^ measures health-related quality of life. We used the EQ-5D visual analogue scale by means of which respondents rate their own current health state on a scale ranging from 0 to 100, with higher values indicating better health.

#### Patient assessment of chronic illness care (PACIC)

The PACIC^60,61^ is an 11-item measure that assesses patients’ evaluations of professional care they have received regarding medical long-term conditions. Using 11-point scales, respondents rate how often they had received or had been satisfied with certain medical interventions during the past six months. Mean scores range from 1 to 11, with higher values indicating better evaluations of medical care.

#### Patient activation measure (PAM)

The PAM^62,63^ consists of 13 items that measure patients’ knowledge, skill, and confidence for health-related self-management. Respondents rate how strongly they agree with related statements using four-point Likert-type scales. Sum scores range from 13 to 52, with higher values indicating better patient activation.

### Data analyses

Data analyses were performed using SPSS (version 27)^64^ and R (version 3.6.3)^65^. We used standard descriptive and non-parametric inferential statistics to analyze the sample characteristics and their relationships to OASIS-D scores. We examined the factor structure for the total sample by running an exploratory factor analysis (EFA) with varimax rotation. Eigenvalues, item factor loadings, and the amount of explained variance are reported. We further performed confirmatory factor analyses (CFAs) using the maximum likelihood (ML) approach to estimate a one-factor model without residual correlations, a one-factor model that allowed for a residual correlation between items 1 and 2, and a two-factor model (with items 1 and 2 loading on one factor and items 3, 4, and 5 loading on another). Model fit was evaluated based on the chi-square test, root mean square error of approximation (RMSEA; cutoff: < 0.05) with 90% confidence interval (CI), standardized root mean residual (SRMR; cutoff: < 0.08), comparative fit index (CFI; cutoff: > 0.90), Tucker–Lewis index (TLI; cutoff: > 0.90), and goodness of fit (GFI; cutoff: > 0.95). Item factor loadings and error variances are reported. We used the ML estimation method mainly for reasons of comparability with previous studies on the OASIS^25^. However, since the items of the OASIS-D can be considered type ordinal in terms of measurement scale, we additionally used the diagonally weighted least squares (DWLS) estimator on the polychoric correlation matrix to test the CFA models described above. The DWLS estimator (as compared to the ML estimator) has been shown to yield more accurate estimates of factor loadings and inter-factor correlations when the observed variables are ordinal^66^.

To assess internal consistency, we calculated Cronbach’s alpha and Guttman’s Lambda 6 for the total sample. To assess retest reliability, we calculated an intraclass correlation coefficient (ICC) assuming a mean-rating (k = 5), absolute-agreement, two-way mixed effects model. To examine convergent and discriminant validity, we calculated bivariate Spearman rank correlations (since several test scores were not normally distributed) between the questionnaires collected from the PDA-subsample. We expected medium to large correlations (> 0.30) of the OASIS-D with BAI, MI, ACQ, PHQ-9, ASI-3, EQ-5D, and number as well as intensity of panic attacks, and low correlations (< 0.30) with PAM and PACIC. All self-report measures showed good to excellent internal consistency in our sample, with Cronbach’s alphas ranging from 0.81 to 0.95 (see Supplementary Table 1, for details).

To determine an optimal clinical cut-score for diagnostic purposes, we calculated ROC curves for the total sample using a criterion measure derived from the PHQ panic module: Patients who responded ‘yes’ to three or four of PHQ’s diagnostic questions were regarded as positive cases and patients who responded ‘yes’ to less than three as negative cases. This scoring algorithm has been shown to detect panic disorder in primary care patients with high sensitivity (86%) and specificity (91%)^67^. We calculated Youden’s index^68^ to find a cut-score that would maximize sensitivity as well as specificity.

To derive a cut-score for individual change, we calculated the Reliable Change Index (RCI)^69^ from the standard deviation and internal consistency of the OASIS-D retest measure. The RCI indicates whether a change score (i.e., a difference in individual OASIS-D scores between any two measurements) exceeds a difference that may have occurred merely due to measurement error. Thus, the RCI-based cut-score indicates the minimal individual difference score that would represent statistically reliable change at a given significance level.

To evaluate whether the data from the total sample fit the Rasch Rating Scale Model^70^, we first calculated infit and outfit chi-square statistics. Here, values close to 1 indicate perfect item fit, values > 1 poor item fit, and values < 1 overfit, with 0.6. to 1.4 being an acceptable range^71^. We further examined person fit, by calculating chi-square based Z-values, and the person separation reliability. Using the Rasch Rating Scale Model, we tested for local item independence by calculating Yen’s Q_3_ statistic that yielded Pearson correlations for each pair of item residuals^72^. We compared each of these correlations to the average item residual correlation. Simulation studies have shown that residual correlations at > 0.2 above the average may indicate local item dependence, and that a residual correlation between independent items at > 0.3 above the average is highly unlikely^73,74^. To examine measurement invariance, we used a recursive partitioning algorithm able to automatically detect subgroups exhibiting DIF in a data-driven way^75^. For the total sample, we investigated DIF as a function of age, gender, and being included in the PDA-subsample. For the PDA-subsample, we investigated DIF as a function of age, gender, and being diagnosed with comorbid agoraphobia and/or comorbid depression. Tree-plots are used to depict noninvariant subgroups in the form of terminal nodes as well as region plots. For each item, region plots visualize the most probable category responses over the range of the latent trait in the detected subgroups.

## Results

For the total sample, the OASIS-D mean score was *M* = 8.08 (SD = 4.80, Median [*Mdn*] = 8, interquartile range [IQR] = 8.00). OASIS-D scores were lower for men (*M* = 7.15, SD = 4.79, *Mdn* = 7, IQR = 8.00) than for women (*M* = 8.59, SD = 4.73, *Mdn* = 9, IQR = 7.00), Mann–Whitney’s *U* = 233,461.50, z = −5.27, *p* < 0.001, *r* = 0.14. The correlation between OASIS-D scores and age was *r*_*s*_ = −0.16, *p* < 0.001. The median number of ‘yes’-answers to the PHQ panic module was 2 (IQR = 4.00). Patients with different numbers of ‘yes’-answers to the PHQ panic module showed differences in OASIS-D scores, Kruskal–Wallis’ *H*(4) = 600.13, *p* < 0.001, with OASIS-D scores being higher for patients with a higher number of ‘yes’-answers, Jonckheere–Terpstra’s *J* = 521,386.50, z = 24.62, *p* < 0.001, *r* = 0.67 (see Supplementary Fig. 1, for descriptive statistics).

For the PDA-subsample, the OASIS-D mean score was *M* = 12.51 (SD = 2.78, *Mdn* = 12, IQR = 4.00). OASIS-D scores were higher for patients with comorbid agoraphobia (*M* = 12.75, SD = 2.78, *Mdn* = 13, IQR = 4.00) than for those without (*M* = 11.75, SD = 2.63, *Mdn* = 12, IQR = 4.00), Mann–Whitney’s *U* = 18,361.50, z = 3.02, *p* = 0.003, *r* = 0.15. Likewise, OASIS-D scores were higher for patients with comorbid depressive disorders (*M* = 13.41, SD = 2.88, *Mdn* = 14, IQR = 4.50) than for those without (*M* = 11.98, SD = 2.57, *Mdn* = 12, IQR = 4.00), Mann–Whitney’s *U* = 25,349.50, z = 4.98, *p* < 0.001, *r* = 0.25. Within the PDA-subsample, OASIS-D scores did not significantly differ with gender, marital status, or education, and were not significantly correlated with age.

### Exploratory factor analysis

Bartlett’s test of sphericity (*p* < 0.0001) and the Kaiser–Meyer–Olkin measure (0.85) indicated that the data were well suited to factor analysis. The EFA revealed only one eigenvalue greater 1 (3.35; next highest eigenvalue: 0.52), suggesting a one-factor solution. The first factor accounted for 66.8% of the variance. Factor loadings were 0.86, 0.85, 0.75, 0.82, and 0.81 for items 1 to 5, respectively.

### Confirmatory factor analyses

**Figure 1:**
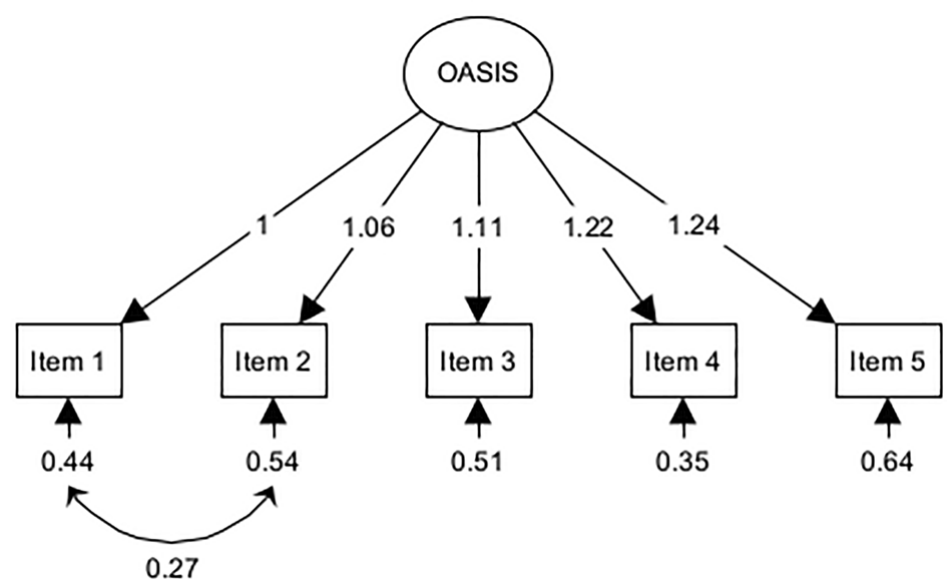
Confirmatory factor analysis for one-factor solution with residual correlation between items 1 and 2. Rectangles symbolize measured variables and the circle the latent construct. Standardized coefficients are given for item factor loadings and error variances.

The one-factor model without residual correlations did not fit the data well, *χ*^2^ = 357.94, *p* < 0.001, RMSEA = 0.23 (90% CI = 0.21–0.25), SRMR = 0.04, CFI = 0.92, TLI = 0.85, GFI = 0.89. The one-factor model that allowed for a residual correlation between items 1 and 2 showed an acceptable fit, *χ*^2^ = 12.98, *p* = 0.01, RMSEA = 0.04 (90% CI = 0.02–0.07), SRMR = 0.01, CFI = 1.00, TLI = 1.00, GFI = 1.00. In this model (Fig. 1), all items displayed significant loadings on the latent factor (all *p*s < 0.001). The residual variances of items 1 and 2 were significantly correlated at 0.27 (*p* < 0.001). The two-factor model also fitted the data well (with fit statistics being identical to those of the one-factor model that allowed for a residual correlation between items 1 and 2) and showed that both factors were correlated at 0.71 (*p* < 0.001). We thus rejected the two-factor model for conceptual reasons: poor discriminating validity of highly overlapping factors. Repeating the CFAs using DWLS (instead of ML) estimation yielded essentially the same results for each of the models (see Supplementary Analysis 1, for details).

### Internal consistency and retest reliability

The internal consistency of the OASIS-D was excellent in the total sample, Cronbach’s α = 0.91 (95% CI = 0.90–0.92), Guttman’s λ-6 = 0.90, and good in the PDA-subsample, Cronbach’s α = 0.81 (95% CI = 0.75–0.86), Guttman’s λ-6 = 0.80. An ICC(A,2) of 0.60 (95% CI = 0.22–0.77) indicated moderate test–retest reliability.

### Convergent and discriminant validity

Table 2 shows bivariate correlations between the self-report measures collected from the PDA-subsample. Suggesting convergent validity, the OASIS-D showed medium to strong correlations with measures of anxiety (BAI, MI, ACQ, ASI-3), depression (PHQ-9), and health-related quality of life (EQ-5D) and was strongly correlated with the number (*r*_*s*_ = 0.52; *p* < 0.001) as well as intensity (*r*_*s*_ = 0.56; *p* < 0.001) of panic attacks. Suggesting discriminant validity, the OASIS-D correlated weakly with measures of patients’ evaluations of medical care (PACIC) and patient activation (PAM).

**Table 2:** OASIS-D correlations with convergent and discriminant validity measures.

	OASIS-D	BAI	MI	ACQ	ASI-3	PHQ-9	EQ-5D	PACIC	PAM
OASIS-D	1	0.66*	0.46*	0.54*	0.42*	0.58*	− 0.43*	− 0.18	− 0.18
BAI		1	0.33*	0.64*	0.44*	0.50*	− 0.40*	− 0.06	− 0.23
MI			1	0.35*	0.26	0.32*	− 0.28*	0.01	− 0.13
ACQ				1	0.55*	0.56*	− 0.29	− 0.13	− 0.15
ASI-3					1	0.61*	− 0.38*	0.08	− 0.04
PHQ-9						1	− 0.46*	− 0.05	− 0.23
EQ-5D							1	0.02	0.11
PACIC								1	0.36*
PAM									1

*ACQ* Anxiety Cognitions Questionnaire, *ASI-3* Anxiety Sensitivity Index-3, *BAI* Beck Anxiety Inventory, *EQ-5D* EuroQoL questionnaire (visual analogue scale), *MI* Mobility Inventory (subscale ‘alone’), *OASIS-D* Overall Anxiety Severity and Impairment Scale (German version), *PACIC* Patient Assessment of Chronic Illness Care, *PAM* Patient Activation Measure, *PHQ-9* Patient Health Questionnaire (depression module).

*Correlation was statistically significant at *p* < 0.001.

### Cut-scores

A ROC curve was calculated for the total sample to identify an appropriate OASIS-D cut-score that would be optimal to determine the presence vs absence of PDA in patients (Supplementary Fig. 2). The area under the curve (AUC) was 0.87 (asymptotic 95% CI = 0.85–0.89), suggesting good classificatory performance. Table 3 lists potentially viable cut-scores, for which both sensitivity and specificity values exceeded 0.5. Based on Youden’s index, we judged a cut-score of ≥ 8 as optimal.

**Table 3:** Characteristics of possible OASIS-D cut-scores for identification of primary care patients with panic disorder with/without agoraphobia.

OASIS-D cut-score	Sensitivity	Specificity	Youden’s index* J*^a^	% correctly classified^b^
≥ 6	0.93	0.56	0.48	73.3
≥ 7	0.90	0.66	0.56	77.4
≥ 8	0.85	0.75	0.60	80.0
≥ 9	0.77	0.82	0.59	79.6
≥ 10	0.71	0.85	0.56	78.3
≥ 11	0.63	0.91	0.54	77.7
≥ 12	0.51	0.95	0.45	74.0

^a^Calculation formula: *J* = sensitivity + specificity − 1.

^b^Overall accuracy computed as the ratio between the number of patients correctly classified as positive or negative cases and the total number of patients.

Using data from the PDA-subsample, we further calculated the RCI to derive a cut-score for reliable individual change. The critical difference was 4.39 at a significance level of α = 0.05 (5.77 at α = 0.01). Thus, a decrease in the OASIS-D score of ≥ 5 would indicate statistically reliable improvement and an increase of ≥ 5 statistically reliable deterioration.

### Rasch model based analyses

We first calculated several fit statistics to assess the extent to which the observed item responses fit the expectations of the Rasch Rating Scale Model. Infit and outfit statistics suggested that all items of the OASIS-D fitted the model well, with values ranging from 0.81 to 1.16 and from 0.81 to 1.14, respectively (Supplementary Table 2). A visual data inspection also suggested that the data were well in accordance with the expected scores (Supplementary Fig. 3). The calculation of person fit indicated that only 3.4% of patients showed substantial deviations from the predicted response pattern (as indicated by Chi-square-based Z-values exceeding 1.96). The person separation reliability was high at 0.87.

#### Local item independence

The range of item residual correlations yielded by the Q_3_ statistic was −0.39 to 0.25 (Supplementary Table 3). The average item residual correlation was −0.22. The residual correlation between items 1 and 2 was the only positive correlation, differing from the average item residual correlation by 0.47. This suggested a local dependence between items 1 and 2. All other correlations were negative and did not markedly diverge from the average item residual correlation, with differences ranging from 0 to 0.21.

#### Measurement invariance

**Figure 2:**
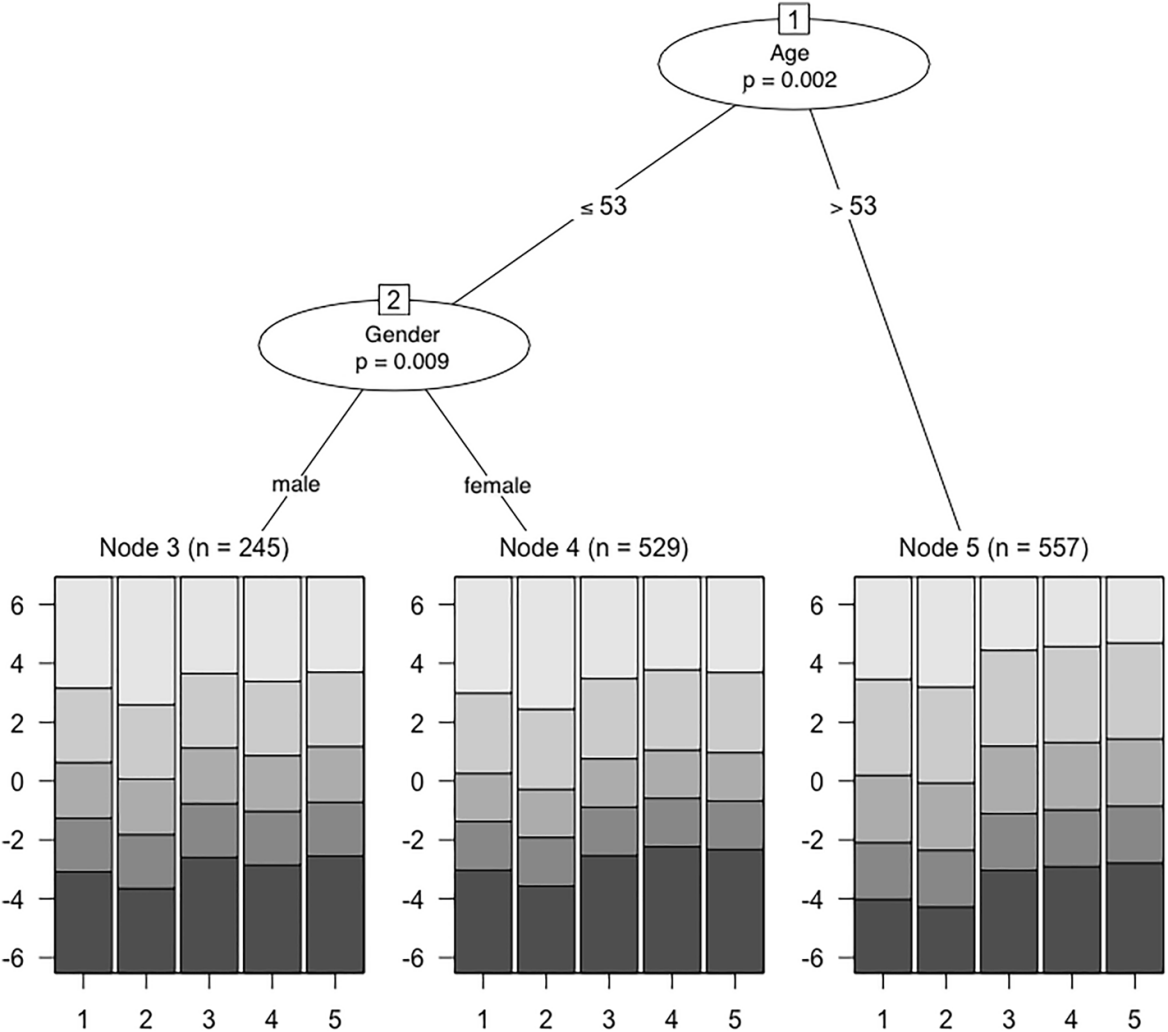
Rating scale tree showing differential item functioning (DIF) within the total sample. Circles symbolize variables associated with DIF. Terminal nodes correspond to subgroups for which the null hypothesis of measurement invariance was rejected. Region plots are depicted below each terminal node to show the respective item threshold parameters.

Within the total sample, DIF was detected as a function of age (with a cut-point at 53 years) and, for patients of 53 years or younger, as a function of gender (Fig. 2). An inspection of the region plots showed that for patients older than 53 years (node 5), responses to the three middle categories were more probable than for younger patients (nodes 3 and 4). Particularly, a higher latent trait was required for older patients to choose the highest categories of items 3, 4 and 5. An interaction of the variables age and gender was detected, indicating that DIF was present between males (node 3) and females (node 4) only within the subgroup of younger patients. The corresponding region plots showed higher threshold parameters for female patients on item 4, suggesting a higher general item difficulty (i.e., a higher latent trait was required for female as compared to male patients to choose a higher item category).

**Figure 3:**
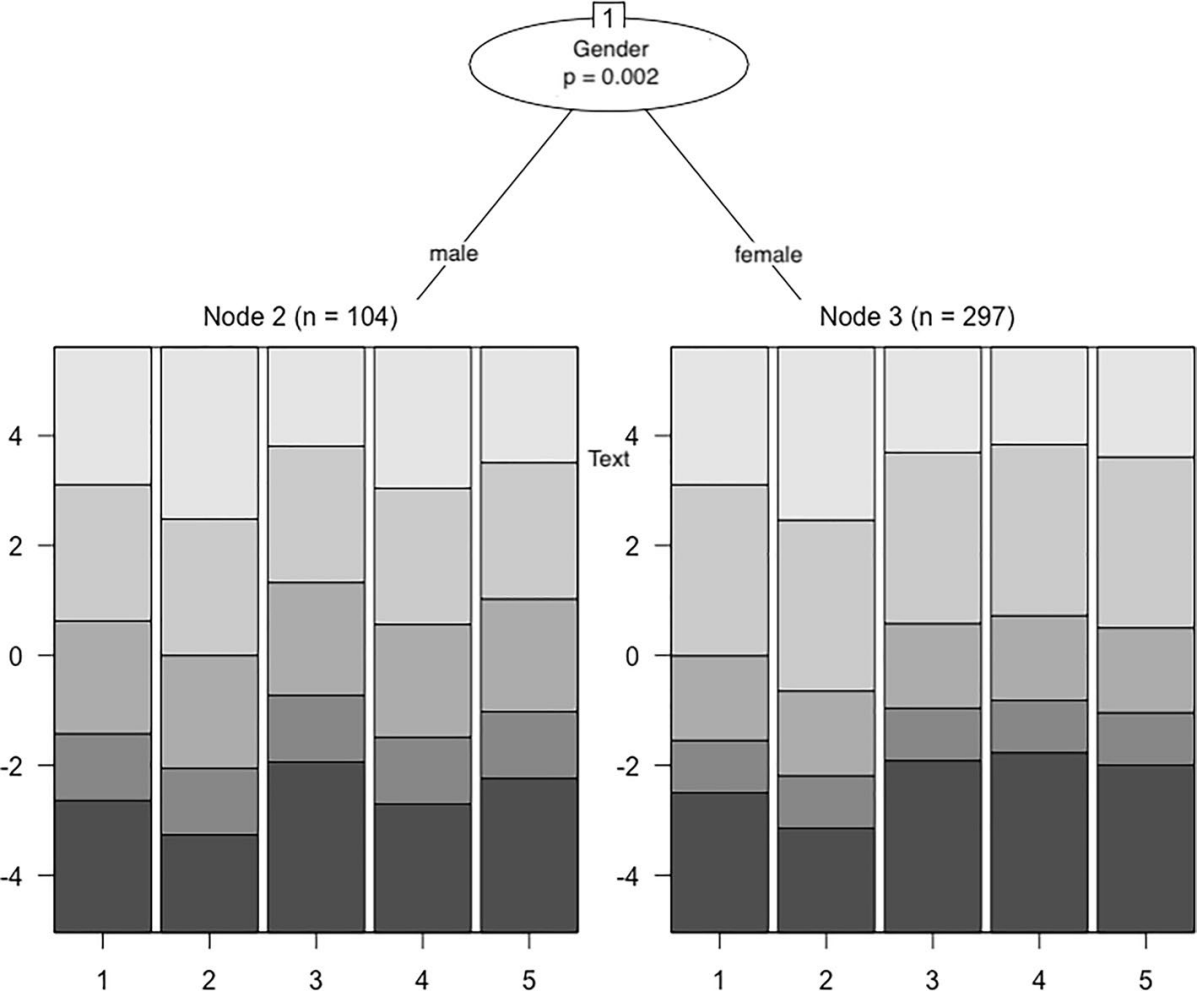
Rating scale tree to assess differential item functioning (DIF) within the subsample of patients with panic disorder with/without agoraphobia. Circles symbolize variables associated with DIF. Terminal nodes correspond to subgroups for which the null hypothesis of measurement invariance was rejected. Region plots are depicted below each terminal node to show the respective item threshold parameters.

Within the PDA-subsample, DIF was detected as a function of gender (Fig. 3). As was the case for the total sample of patients, the estimated threshold parameters of item 4 were higher for female (node 3) than for male patients (node 2), suggesting differences in the general item difficulty. The region plots further indicate that across all items, responses to the second highest category were slightly more probable for female than for male patients.

## Discussion

This study presented and evaluated a German version of the OASIS, using a large convenience sample of primary care patients. While previous studies have relied on statistical methods based on classical test theory, the current study additionally used polytomous extensions of the Rasch model to investigate the OASIS-D.

The EFA clearly pointed to a one-factor solution as was consistently the case in prior investigations of the OASIS^23,25,30,33^. Also like in previous studies^24,25,28,32,35,36^, CFAs revealed that a single-factor model fitted the data only when the residual variances of the first two items were allowed to correlate. Confirming the CFA results, the Rasch model based Q_3_ statistic indicated that items 1 and 2 were most likely not independent. Thus, the current study adds to the evidence that these two items systematically share error variance that does not help to measure the targeted construct but nevertheless influences the test score.

It has been argued that covariance between items 1 and 2 is theoretically plausible because the responses are partly contingent on each other (i.e., if frequency of anxiety is rated zero, severity should also be rated zero)^25,28,34^. Following this explanation, a method effect induces a so-called response dependency between these two items^74^. Method-induced response dependency may affect the estimation of person parameters under the Rasch model and can lead to biased estimates of reliability but should not be considered an important violation of a measure’s unidimensionality^73,76^. Another possible explanation for the residual correlation of items 1 and 2 is that these two items measure something that slightly differs from what the other items measure. For example, items 1 and 2 measure the actual extent of clinical symptoms by asking for frequency and intensity of anxiety or fear, whereas items 3 through 5 measure broader clinical aspects of anxiety disorders by asking for avoidance behavior and interferences in occupational as well as social areas of life. This would lead to a higher similarity of responses to items 1 and 2 as compared to the similarity of responses to items 1 or 2 on the one hand and responses to items 3, 4, or 5 on the other hand. Since a systematically “higher similarity” of responses to items 1 and 2 cannot be explained by a single latent variable, a residual correlation between these items would result when specifying a one-factor model. According to our data, it still makes little sense to assume a two-factor model (with items 1 and 2 loading on one factor and items 3, 4, and 5 loading on another) since we found a high correlation—and thus, poor discriminant validity—between the two factors.

The internal consistency of the OASIS-D was good to excellent, with Cronbach’s alpha falling within the range of values observed by prior studies on the OASIS^23–28,30–33,35,36^. The test–retest reliability was slightly lower than in previous studies^30,31,35^, which is probably due to method effects that may have led to an underestimation of the test–retest reliability in our study: At the first measurement, the variance of OASIS-D scores was constrained by the inclusion criteria of the interventional trial in which the current study was embedded (i.e., only patients showing an OASIS-D score of above seven were included in the PDA-subsample)^39^.

As expected, we found evidence for convergent validity of the OASIS-D with established self-report measures of anxiety, depression, and health-related quality of life as well as for discriminant validity with self-report measures of conceptually distinct constructs. Significant correlations of the OASIS with measures of depression have also been found by prior studies^26,28,30,32,35^. This lack of a clear distinction between anxiety and depression must not necessarily be viewed as a limitation of the measure. Rather, it may reflect that both disorders share important features such as distress or negative affectivity, genetic predisposition, and common neurobiology^77,78^. Furthermore, it must not limit the use of the OASIS-D in primary care settings where it is important to quickly identify patients affected by any psychopathology and where initial pharmacologic treatments would likely be similar for patients with anxiety disorders or depression^26^. A clinical implication of this finding is that patients scoring high on the OASIS-D should be carefully examined for anxiety as well as for depressive disorders.

Some studies have demonstrated that the OASIS is sensitive to therapy-induced changes of symptom severity, which can be interpreted as further evidence for the measure’s construct validity^26,28,32,36^. An investigation of sensitivity to change was not part of the current study. However, the OASIS-D has already been used to monitor patients with PDA receiving an exposure-based intervention in primary care^38^. During this intervention, OASIS-D scores declined by an average of 6.24 points, indicating sensitivity to change^79^. Furthermore, growth mixture modeling of change trajectories suggests that using the OASIS-D for clinical monitoring may help to quickly identify patients who do not respond to the intervention^80^.

Based on ROC analyses, we found that an OASIS-D score of ≥ 8 may serve as an optimal cut-score when screening primary care patients for the presence of PDA. This result is well in line with previous studies on the OASIS^24,25,28,32^ but should however be viewed with caution since we determined patients’ diagnostic status using the PHQ panic module. Although the PHQ has been designed as a diagnostic tool for DSM-IV disorders^45^ and validated for primary care settings^67,81^, this may have introduced method effects. Thus, our results on the optimal cut-score for screening purposes should be confirmed by future studies using full-length structured clinical interviews for diagnostics.

The RCI indicated that an individual difference in OASIS-D scores of ≥ 5 can be considered statistically reliable clinical change. Our results on the RCI closely resemble those found for the original English version^28^ and slightly diverge from those found for the Dutch version of the OASIS^31^. It should be noted that an RCI-derived cut-score is not based on any clinical considerations but calculated from a measure’s observed reliability and standard deviation. Probably because the OASIS-D retest measure showed a lower reliability than the Dutch version, the critical difference was slightly larger in our study.

Rasch model based analyses of measurement invariance detected DIF as a function of age as well as gender within the total sample and as a function of gender within the PDA-subsample. This suggest that the ways in which the items of the OASIS-D are linked to the construct of anxiety severity and impairment systematically differ between older and younger patients as well as between male and female patients. We can only speculate on the specific reasons for DIF. Over the range of the latent trait, the probability of responses to the highest categories of items 3 through 5 was lower for patients older than 53 years than for younger patients. Hypothetically, older versus younger patients may differ in their evaluations of avoidance and occupational/social impairments (e.g., because older patients are more likely to be retired). With respect to gender, we found that a higher value of the latent trait was required for female than for male patients to choose higher response categories on item 4. Hypothetically, anxious men may be more likely to fail sociocultural expectations than anxious women, which may particularly be reflected by responses to item 4. We did not find DIF associated with the diagnoses of PDA, agoraphobia, or depression. These null findings must not be interpretated as evidence of absence as they could just as well be due to limited statistical power or other reasons.

One study has examined the original OASIS for DIF using a CFA model that incorporated covariate effects^28^. The authors have interpreted their findings as initial support for measurement invariance with respect to gender but recommended similar analyses in broader samples (e.g., primary care patients) since they have found a potential (nonsignificant) instance of DIF. Two other studies have investigated the Czech version of the OASIS for measurement invariance by means of multi-group CFAs^34,35^. In both studies, evidence was found that men and women responded similarly to the OASIS. These earlier findings on measurement invariance thus diverge from ours, which may be due to differences in sample characteristics, sample size, or statistical analyses.

The current study has meaningful strengths. It is the first that examines the psychometric properties of the OASIS using statistical methods based on probabilistic test theory. Furthermore, this study is the first to examine the OASIS in a diverse sample of patients recruited in small primary care practices. Such practices play a major role in the provision of health care in many countries^82^. Thus, our findings may generalize to naturalistic general medical settings, which may help to facilitate guideline-concordant recognition, diagnostics, and initial treatment of anxiety disorders in primary care^15,16^. For example, our results support the use of the OASIS-D as a screening questionnaire for primary care patients suspected of suffering from clinically relevant levels of anxiety. Patients scoring high on the OASIS-D should be examined by their primary care physicians for anxiety as well as for mood disorders. Furthermore, the OASIS-D may be used to effectively monitor treatment progress, whereby the RCI-derived cut-off may help to determine reliable clinical changes in anxiety severity and related functional impairments. The questionnaire can reliably be administered and scored by medical assistants^79,83^, which is of concern considering primary care physicians’ time-constraints^22^.

This study also has limitations. Patients were recruited within the scope of an interventional clinical trial. This may have introduced a selection bias (i.e., patients with suspicion of anxiety disorders may have been more likely to be screened) that potentially limits the generalizability of results. Moreover, patients were screened only for PDA but not for other mental disorders, and comorbidity with other anxiety disorders was not determined. Although the primary care physicians were trained in diagnostics of PDA and used ICD-10 checklists, clinical diagnoses may have been less reliable than in previous studies. We believe that this does not necessarily limit the validity of our results since a high degree of individual variability is immanently found in naturalistic clinical settings, and the OASIS has been designed as a transdiagnostic measure of anxiety. As with prior evaluations of the OASIS, the current study relied on self-report measures to assess convergent/discriminant validity and optimal cut-off score. This may have introduced method effects as discussed above. The number of variables analyzed for DIF was limited. Like any covariate-based approach, the recursive partitioning algorithm can only detect non-invariance if the relevant variables are available. The Rasch analyses were based on the Rating Scale Model that parsimoniously assumes discrimination parameters and category thresholds to be equal across items. Since this assumption can be violated, it would be worthwhile to perform Rasch analyses based on a 2-parameter logistic model (e.g., the generalized Partial Credit Model) able to estimate item-specific discrimination parameters and item-specific category thresholds. Future studies that wish to follow this approach should collect even larger data sets than we did to prevent convergence issues (a more flexible 2-parameter logistic model requires considerably more parameters to be estimated than the Rating Scale Model). Finally, all results of this study need replication, which is particularly true for the Rasch analyses that are unique to it.

To conclude, this study adds to the growing evidence for the transcultural validity of the OASIS. The German version has demonstrated adequate psychometric properties in a convenience sample of primary care patients. The brevity of the scale may facilitate the recognition, diagnosis, and monitoring of anxiety disorders in naturalistic general medical settings. Because initial evidence for measurement non-invariance was found, caution is warranted when using the scale to compare groups that differ in age or gender.

## Supplementary Information

Supplementary Information.

## Data Availability

The data that support the findings of this study are part of the interventional clinical trial “Jena-PARADISE” (German Clinical Trials Register: DRKS00004386; https://drks.de/search/en/trial/DRKS00004386; Principal Investigator: Jochen Gensichen). Due to legal regulations, the data are not publicly accessible. However, the data and the study code can be obtained from the corresponding author upon reasonable request.
