# Supplementary material for: Psychometric evaluation and Rasch analyses of the German Overall Anxiety Severity and Impairment Scale (OASIS-D)
Source: Sci Rep. 2023 Apr 26;13:6840. doi: 10.1038/s41598-023-33355-0 (PMC10133318; doi:10.1038/s41598-023-33355-0)
Supplement: Supplementary file 1 — Supplementary Information. [file 41598_2023_33355_MOESM1_ESM.pdf]

Psychometric evaluation and Rasch analyses of the German Overall Anxiety Severity and Impairment Scale (OASIS-D)

by

Thomas S. Hiller, Sabine Hoffmann, Tobias Teismann, Karoline Lukaschek, Jochen Gensichen

# – Supplementary information –

Contents:

Supplementary Appendix 1. Overall Anxiety Severity and Impairment Scale, German version (OASIS-D) 2

Supplementary Table 1. Internal consistency (Cronbach's alpha) of self-report measures 3

Supplementary Table 2. Infit and outfit statistics for the Rasch Rating Scale Model 4

Supplementary Table 3. Q_3 statistic (Pearson correlations of item residuals) 4

Supplementary Fig. 1. OASIS-D scores by numbers of positive answers to the PHQ panic module 5

Supplementary Fig. 2. Receiver operating characteristic (ROC) curve for OASIS-D scores to predict the presence of panic disorder with/without agoraphobia 6

Supplementary Fig 3. Observed and expected score curves for the Rasch Rating Scale Model 7

Supplementary Analysis 1. Confirmatory factor analyses using diagonally weighted least squares (DWLS) estimation 8

Supplementary Appendix 1.

Overall Anxiety Severity and Impairment Scale, German version (OASIS-D).

Fragebogen: OASIS-D		
Die folgenden Fragen beziehen sich auf Angst und Furcht.<br>Bitte kreuzen Sie bei jeder Frage diejenige Antwort an, die am besten beschreibt, wie es Ihnen **in der vergangenen Woche** ergangen ist.		
1)	Wie oft hatten Sie Angst?	☐ Nie<br>☐ Selten<br>☐ Gelegentlich<br>☐ Oft<br>☐ Immer
2)	Wenn Sie Angst hatten, wie stark bzw. intensiv war diese Angst?	☐ Gar nicht / Keine Angst<br>☐ Kaum<br>☐ Mittelmäßig<br>☐ Ziemlich<br>☐ Extrem
3)	Wie oft vermieden Sie Situationen, Orte, Dinge oder Aktivitäten aufgrund von Angst oder Furcht?	☐ Nie<br>☐ Selten<br>☐ Gelegentlich<br>☐ Oft<br>☐ Immer
4)	Wie sehr beeinträchtigten Angst oder Furcht Ihre Fähigkeit, notwendige Aufgaben bei der Arbeit, in der Schule oder zuhause zu erledigen?	☐ Gar nicht<br>☐ Kaum<br>☐ Mittelmäßig<br>☐ Ziemlich<br>☐ Extrem
5)	Wie sehr beeinträchtigten Angst oder Furcht Ihre sozialen Aktivitäten und Beziehungen?	☐ Gar nicht<br>☐ Kaum<br>☐ Mittelmäßig<br>☐ Ziemlich<br>☐ Extrem

Supplementary Table 1.

Internal consistency (Cronbach's alpha) of self-report measures.

Measure	Cronbach's alpha (95% confidence interval)
BAI	0.91 (0.88 to 0.92)
MI	0.95 (0.94 to 0.96)
ACQ	0.84 (0.79 to 0.88)
ASI-3	0.91 (0.89 to 0.93)
PHQ-9	0.86 (0.84 to 0.88)
PACIC	0.88 (0.86 to 0.90)
PAM	0.81 (0.76 to 0.86)

Abbreviations: ACQ, Anxiety Cognitions Questionnaire; ASI-3, Anxiety Sensitivity Index-3; BAI, Beck Anxiety Inventory; MI, Mobility Inventory (subscale 'alone'); OASIS-D, Overall Anxiety Severity and Impairment Scale (German version); PACIC, Patient Assessment of Chronic Illness Care; PAM, Patient Activation Measure; PHQ-9, Patient Health Questionnaire (depression module)

Supplementary Table 2.

Infit and outfit statistics for the Rasch Rating Scale Model.

	Infit	Outfit
Item 1	0.81	0.81
Item 2	0.93	0.92
Item 3	1.16	1.14
Item 4	1.00	0.96
Item 5	1.01	0.98

Supplementary Table 3.

Q_3 statistic (Pearson correlations of item residuals).

	Item 1	Item 2	Item 3	Item 4	Item 5
Item 1	1	.25	-.39	-.30	-.37
Item 2		1	-.33	-.32	-.36
Item 3			1	-.22	-.13
Item 4				1	-.01
Item 5					1

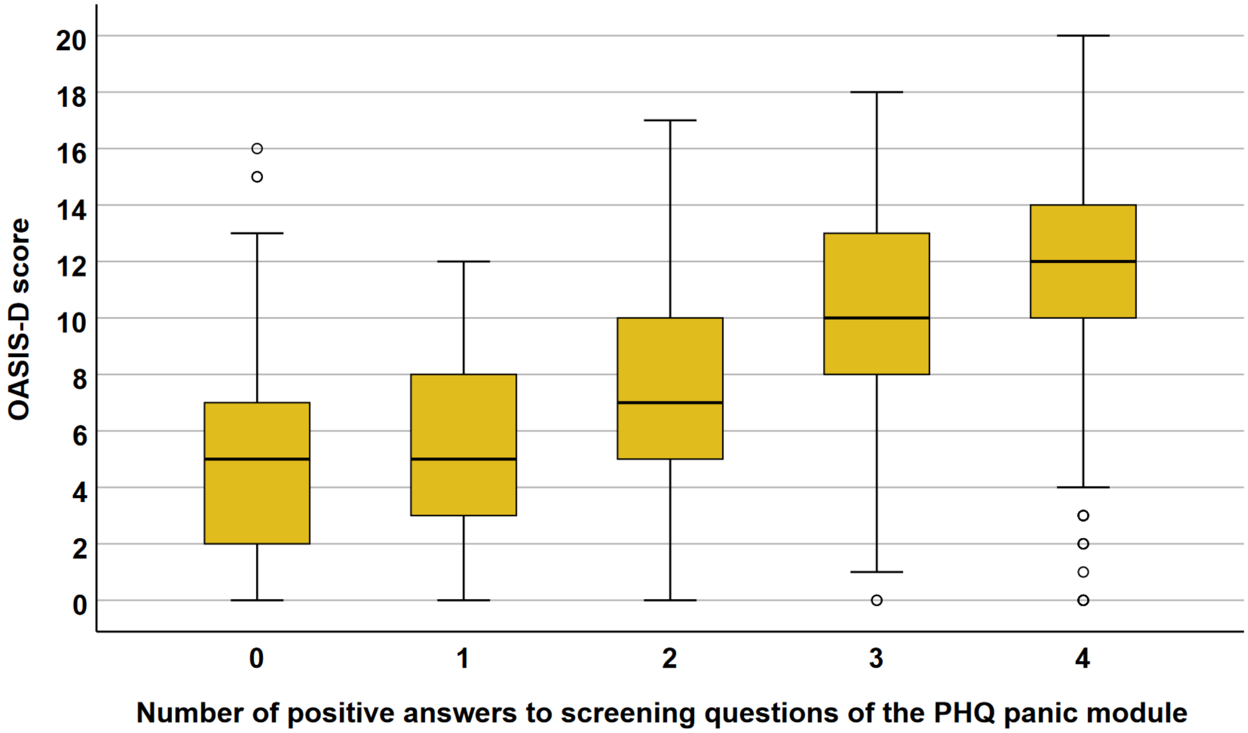

Supplementary Fig. 1.

OASIS-D scores by numbers of positive answers to the PHQ panic module. For each possible number of positive answers to the PHQ panic module, the corresponding distributions of OASIS-D scores are plotted using Box-Whisker diagrams. Within each diagram, the thick line at the center represents the median, the box shows the interquartile range (i.e., the middle 50% of scores), the whiskers indicate the top and bottom 25% of scores (except for outliers), and circles represent outliers. Due to missing data points on the PHQ panic module or on the OASIS-D, 41/1398 (2.9%) patients were excluded from this analysis. Case numbers were n = 533, n = 60, n = 118, n = 214, and n = 432, for 0 to 4 positive answers to the PHQ panic module, respectively. Abbreviations: OASIS-D, Overall Anxiety Severity and Impairment Scale (German version); PHQ, Patient Health Questionnaire.

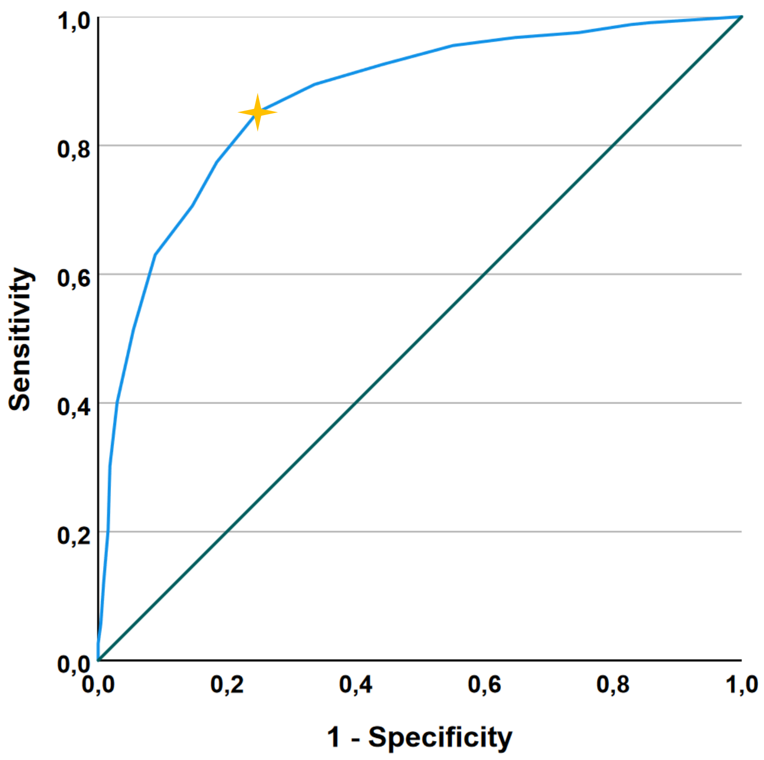

Supplementary Fig. 2.

Receiver operating characteristic (ROC) curve for OASIS-D scores to predict the presence of panic disorder with/without agoraphobia. The cut-score of ≥8 (symbolized by ✦) was located at the point of the ROC curve that was closest to the top left corner of the diagram, indicating the best possible performance. Abbreviations: OASIS-D, Overall Anxiety Severity and Impairment Scale (German version).

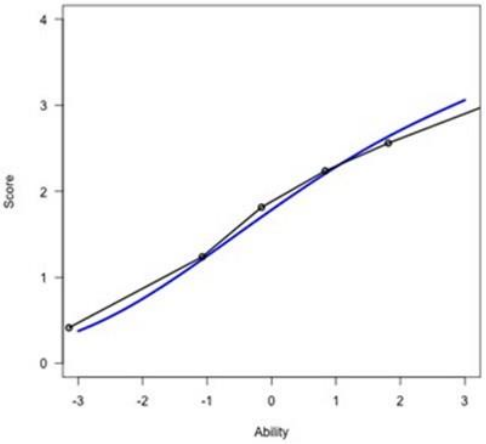

(a) Item 1

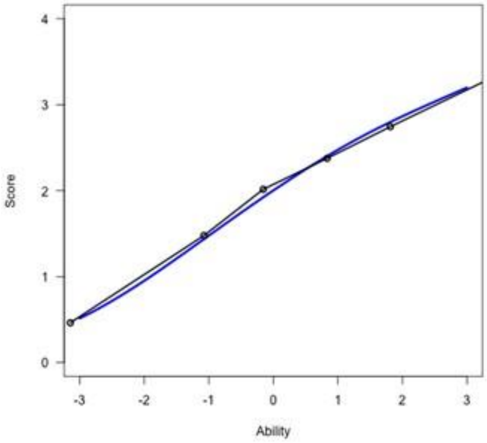

(b) Item 2

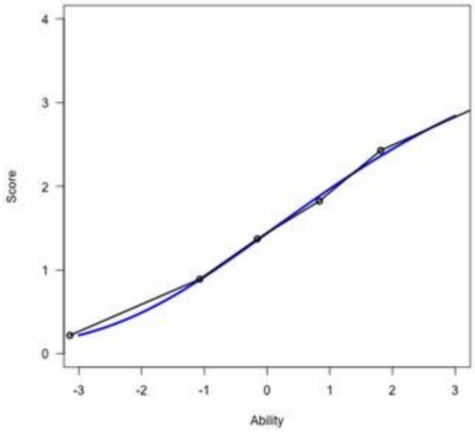

(c) Item 3

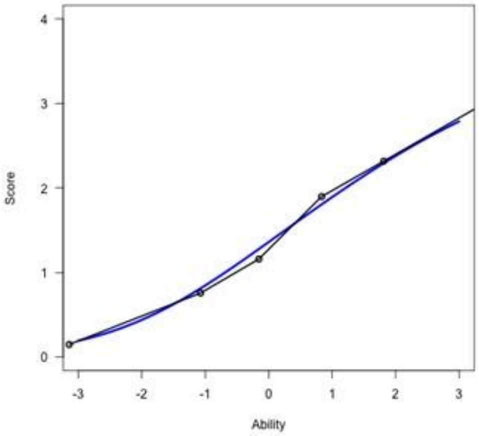

(d) Item 4

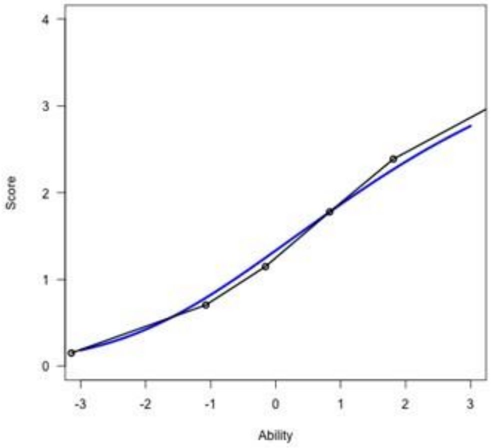

(e) Item 5

Supplementary Fig 3.

Observed and expected score curves for the Rasch Rating Scale Model. The black dots/lines indicate the observed data and the blue curves the characteristic expected item curves.

Supplementary Analysis 1.

Confirmatory factor analyses using diagonally weighted least squares (DWLS) estimation.

To examine the factor structure of the OASIS-D, we have performed confirmatory factor analyses (CFAs) using the maximum likelihood (ML) estimator. Results of these analyses support a one-factor model with residual correlation between items 1 and 2. Since the items of the OASIS-D can be considered type ordinal (instead of type interval or higher) in terms of measurement scale, we performed a recalculation of the CFAs using the diagonally weighted least squares (DWLS) estimator on the polychoric correlation matrix (instead of the ML estimator on the sample covariance matrix).

When using the DWLS estimator, the one-factor model without residual correlations did not fit the data well, $\chi 2 = 37.68$, $p < 0.001$, RMSEA = 0.07 (90% CI = 0.05 to 0.09), SRMR = 0.04, CFI = 0.92, TLI = 0.85, GFI = 0.89. The one-factor model that allowed for a residual correlation between items 1 and 2 showed an acceptable fit, $\chi 2 = 1.54$, $p = 0.82$; RMSEA = 0 (90% CI = 0 to 0.02), SRMR = 0.01, CFI = 1, TLI = 1, GFI = 1. In this model (Fig. A1), all items displayed significant loadings on the latent factor (all *p*s < 0.001). The residual correlation between items 1 and 2 was 0.27 ($p < 0.001$). The two-factor model also fitted the data well (with fit statistics being identical to those of the one-factor model that allowed for a residual correlation between items 1 and 2) and showed that both factors were correlated at 0.71 ($p < 0.001$). We thus rejected the two-factor model for conceptual reasons: poor discriminating validity of the two factors. To conclude, the additional CFAs with DWLS estimation confirm the results that have been obtained from the original CFAs with ML estimation, supporting a one-factor model with residual correlation between items 1 and 2.

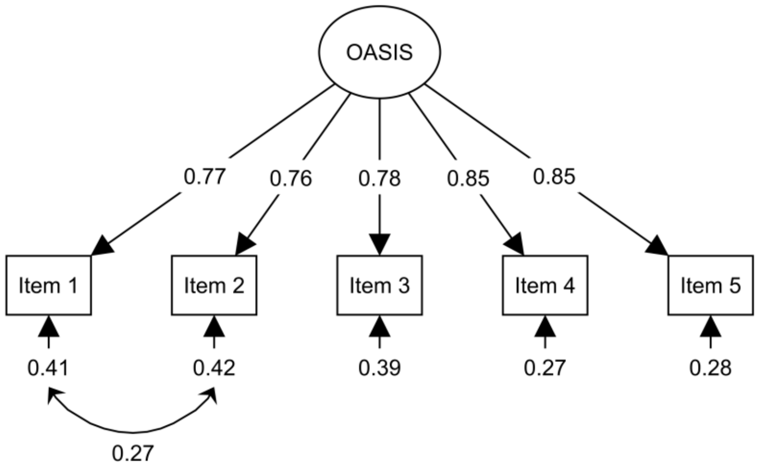

Figure A1. Confirmatory factor analysis with diagonally weighted least squares (DWLS) estimation based on the polychoric correlation matrix. Rectangles symbolize measured variables and the circle the latent construct. Standardized coefficients are given for item factor loadings and error variances.
